# Perseverative Responding in Nigerian Chronic Alcohol and Marijuana Users

**DOI:** 10.1080/10826084.2020.1725055

**Published:** 2020-02-13

**Authors:** Tochukwu Nweze, Cyprian C. Eze, Florian Lange

**Affiliations:** a Department of Psychology, University of Nigeria, Nsukka, Nigeria; b MRC Cognition and Brain Sciences Unit, University of Cambridge, Cambridge, UK; c Behavioral Engineering Group, KU Leuven, Leuven, Belgium

**Keywords:** Executive function, alcohol, marijuana, Wisconsin Card Sorting Test

## Abstract

**Background:**

Chronic consumption of alcohol and marijuana, especially when initiated at an early age, has been implicated in cognitive alterations in the domain of executive functioning. Despite the robustness of this finding in Western populations, its generalizability to other cultural contexts is largely unknown. In this study, we examined whether the regular use of alcohol or marijuana use relates to impaired executive functioning in male students of a Nigerian university.

**Methods:**

Chronic alcohol users (*n*=39), chronic marijuana users (*n*=35) and drug-abstinent control participants (*n*=40) recruited through snow-ball sampling technique completed a computerized version of the Wisconsin Card Sorting Test (cWCST). As an established measure of executive functioning, the cWCST allows for the simultaneous assessment of three distinct executive processes: set shifting, rule inference, and set maintenance. Results revealed a selective set-shifting deficit in both alcohol and marijuana users.

**Results:**

Both groups committed significantly more perseverative errors than the control group, and group differences were significantly stronger on this indicator of set shifting than on indicators of rule inference or set maintenance.

**Conclusions:**

Our findings support the generalizability of drug-related deficits in executive functioning and contribute to the characterization of executive dysfunction in non-Western populations. Future longitudinal studies are required to clarify whether executive dysfunction is an antecedent or consequence of alcohol and marijuana use in young Nigerians.

## Introduction

Alcohol and marijuana are the two most commonly used drugs among adolescents and emerging adults (Johnston, Bachman, O’Malley, & Schulenberg, 2010). The chronic consumption of alcohol and marijuana is fraught with serious deleterious effects, especially when initiated early in life (Ernst & Korelitz, 2009). One of the best established neuropsychological consequences of alcohol and marijuana use is impairment in executive functioning. Executive functions are higher-level cognitive processes that enable adaptive, goal-directed behavior by exerting control over lower-level functions (Diamond, 2013).

Chronic use of alcohol and marijuana seems to relate to alterations in executive functioning and its neural correlates (Crane, Schuster, Fusar-Poli, & Gonzalez, 2013; Solowij & Pesa, 2010). For example, chronic alcohol (Goldstein et al., 2004) and marijuana users (Pope et al., 2003) have been found to show performance deficits on the Wisconsin Card Sorting Test (WCST; Berg, 1948). However, performance on neuro-psychological tests (such as the WCST) depends on multiple functions, executive and non-executive (Lange, Seer, & Kopp, 2017). This non-specificity problem can partly be addressed by distinguishing between different types of errors on a computerized version of the WCST (cWCST, Lange, Kröger, et al., 2016; Lange, Seer, Dengler, Dressler, & Kopp, 2016).

In the present study, we administered the cWCST to simultaneously assess the executive domains of set shifting, rule inference, and set maintenance in chronic alcohol and marijuana users from Nigeria. To our knowledge, this is the first examination of executive functioning in users of these drugs in sub-Saharan Africa. Most previous studies in the field have been conducted in Western and Asian countries and it is unclear whether these results can be generalized to the Nigerian population. The cWCST data collected in this study address this question of generalizability and provide first insights into the profile of executive deficits in Nigerian alcohol and marijuana users.

## Methods

### Participants

Participants for the study consisted of 114 male college students (age range: 19-35 years; *M* = 24.89; *SD* = 2.54) recruited through snow-ball sampling technique from the university town Nsukka in south-east Nigeria. Participants were included in one of three groups. Chronic marijuana users (*n*=35; *M*_age_ = 25.40; *SD* = 2.53) self-reported to smoke at least five grams of marijuana per week (*M* = 10.40; *SD* = 6.04) and they reported not to drink more than four bottles of beer (or equivalent amounts of other alcoholic beverages) per week. Chronic alcohol drinkers (*n* = 39; *M*_age_ = 25.05; *SD* = 2.29) self-reported to drink at least five bottles of beer per week (*M*=6.36; *SD* = 1.98) and not to smoke at least five grams of marijuana per week. Controls (*n*=40; *M*_age_ = 24.20; *SD* = 2.68) self-reported to have abstained from alcohol use at least for the past one year, to have never smokedmarijuana in their lifetime, and to have consumed no more than five bottles of alcoholic beverages in their lifetime. The three groups did not differ significantly with regard to age, *F*(2, 111) = 2.31, *p* = .104, and education, *F*(2, 111) = 1.83, *p* = .166. All procedures were in accordance with the Declaration of Helsinki and approved by the local ethics committee.

### Procedures and assessment

Eligible participants were approached and scheduled for computerized assessment at the nearest convenient laboratory or library in the University. Study objectives were explained in a consent form, which participants signed prior to participation. All participants completed the latest version of the cWCST (described in detail by Lange & Dewitte, 2019, and available for download at https://osf.io/5t6fs/). On each trial of the cWCST, participants were required to match cards according to one of three possible sorting rules. Card sorts were followed by a feedback cue that indicated whether the applied sorting rule should be repeated or switched. Rules changed after runs of two or more repetitions. Participants completed six practice runs and 42 task runs or a maximum of 250 trials, whichever occurred first.

Following established procedures (Lange, Kröger, et al., 2016), we analyzed three types of errors (perseveration, integration, set-loss) committed on three cWCST trials types (switch, integration, repetition) to distinguish three facets of executive functioning (set shifting, rule inference, set maintenance). Mean response times (RT) for correct responses on these trials (after filtering of RT < 200ms and >3SD above the mean) were analyzed as a secondary outcome measure. cWCST performance measures were compared between groups using mixed ANOVAs with Greenhouse-Geisser correction. By this means, it was possible to not only examine whether groups of alcohol users, marijuana users, and controls differed in overall cWCST performance, but also whether group differences were specific to a particular cWCST performance measure. The level of significance was set at *α* = .05. All data, analysis scripts, and a more detailed description of the methods can be found at https://osf.io/z9h3j/. (see also Supplementary Materials).

## Results

**Figure 1:**
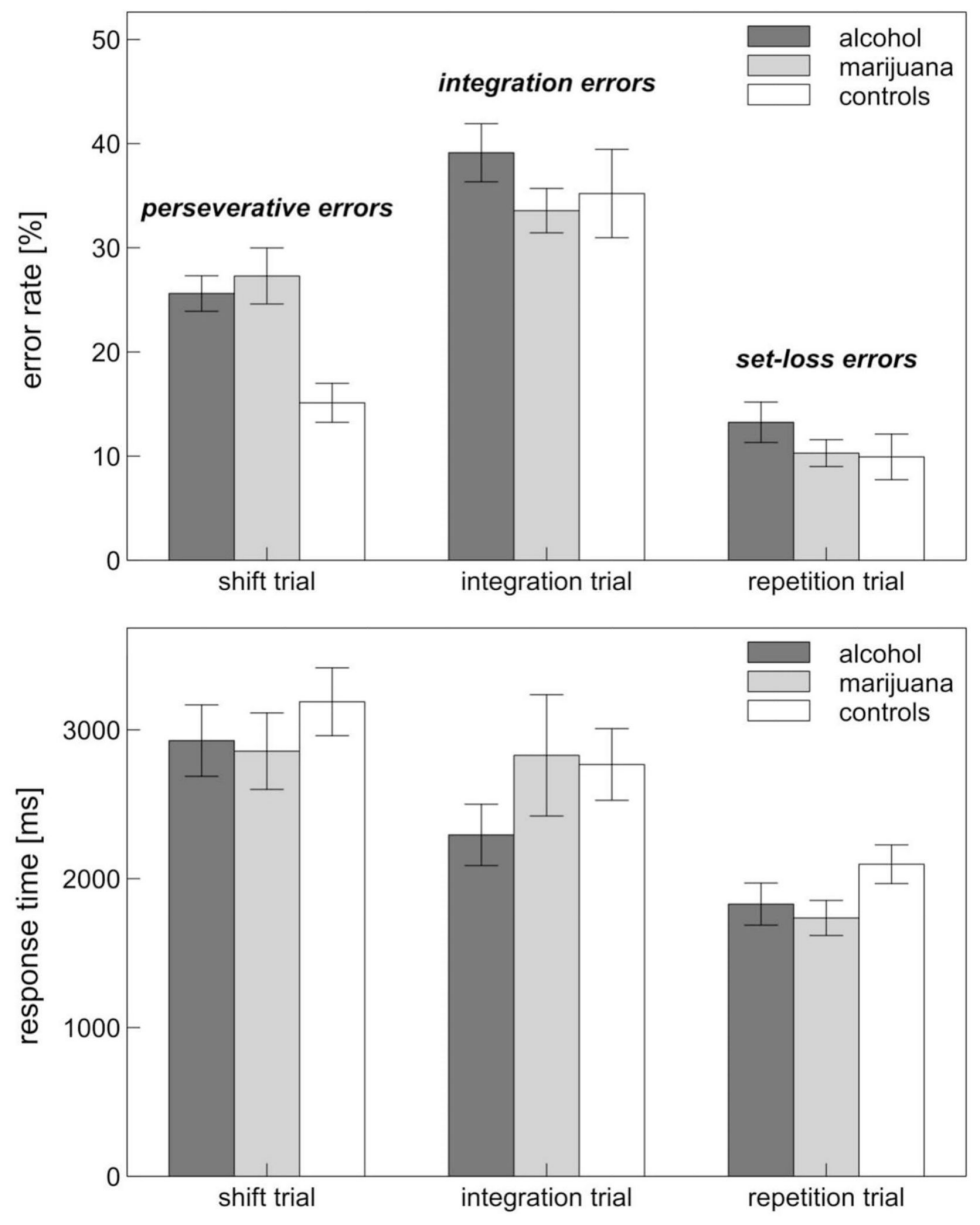
Accuracy and latency of participants’ responses to different trials of the computerized Wisconsin Card Sorting Test. Error bars indicate standard errors of the mean.

Error rates on the cWCST were subjected to a 3×3 ANOVA involving the within-subject factor Error Type (perseveration, integration, set-loss) and the between-subjects factor Group (alcohol, marijuana, control). The main effect of Group did not reach significance, *F*(2, 111) = 3.05, *p* = .051, η^2^ = .052, but the interaction between Group and Error Type did, *F*(3.57, 197.91) = 2.93, *p* = .027, η^2^ = .050 (see Figure 1). Bonferroni-adjusted simple effects tests showed that both alcohol users (*p* = .001) and marijuana users (*p* < .001) committed significantly more perseverative errors than controls. In contrast, no group differences were observed with regard to the other two error types (all *p* > .623). Simple effects tests did not show any significant differences between the two drug groups on any error measure (all *p* > .833). The same analysis strategy was repeated using mean response times as the outcome variable. A Trial Type (switch, integration, repetition) × Group ANOVA did not reveal a significant main effect of Group, *F*(2, 111) = 0.92, *p* = .402, η^2^ = .016. The Trial Type × Group interaction was not significant either, *F*(3.71, 206.03) = 1.00, *p* = .404, η^2^ = .018.

## Discussion

A fine-grained analysis of cWCST error profiles revealed that chronic alcohol and marijuana users committed significantly more perseverative errors than controls. This performance measure was significantly more affected than other error scores, thus pointing to a selective set-shifting deficit in chronic alcohol and marijuana users.

The result from our study is consistent with similar studies with western population samples (Fontes et al., 2011; Gruber, Sagar, Dahlgren, Racine, & Lukas, 2012; Lane, Cherek, Tcheremissine, Steinberg, & Sharon, 2007; Pope et al., 2003). In contrast to these studies, we were able to demonstrate that drug-use-related cWCST deficits were largely specific to the domain of cognitive set shifting (as indexed by the number of perseverative errors). Compared to drug-abstinent controls, young Nigerian alcohol and marijuana users seem to have relatively circumscribed deficits in this domain of executive functioning.

There are several possible explanations for the differences observed between drug-consuming individuals and control participants. One possibility is that continued exposure of marijuana and alcohol has over time resulted in neural alterations that manifest themselves as set-shifting deficits on the cognitive level. Many of our participants reported to have started using substances at a very early age where the brain might be particularly susceptible to these substances (Fontes et al., 2011; Pope et al., 2003). Alternatively, the large number of perseverative errors committed by alcohol and marijuana user might be an indicator of a premorbid cognitive deficit predating the onset drug use. Longitudinal studies are needed to test these possibilities. An ideal investigation would assess participants at different stages of childhood development, before onset of alcohol and marijuana use, followed by additional assessments in adolescence and young adulthood after drug-use onset, while controlling other risk factors of substance use.

The results of this study should be interpreted in the light of some limitations. First, the cross-sectional design of this study has limited our ability to make conclusions about the direction of the relationship between set-shifting deficits and alcohol and marijuana use. Second, while our groups were reasonably matched with regard to age and education, there could have been other variables not taken into account. For example, participants were asked if they had abstained from alcohol and marijuana use for at least 12 h prior to participation, but due to logistics, no urinalysis was done for confirmation. Additionally, only males were recruited for the study, thus precluding generalization to the female population. Future studies might benefit from addressing these limitations to further elucidate the relationship between executive functioning and drug use.

In conclusion, this study revealed specific perseverative tendencies on the cWCST in alcohol and marijuana users that are suggestive of a deficit in cognitive set shifting. By confirming the findings of previous studies from Western countries, our results indicate that alcohol- and marijuana-related executive impairment generalizes to the Nigerian population. As such, our study illustrates the value of studying the cognitive correlates of drug use in non-Western cultures as well as the use of a fine-grained analysis of executive functioning.

## Supplementary Material

Supplementary materials
