# Supplementary material for: Perseverative Responding in Nigerian Chronic Alcohol and Marijuana Users
Source: Subst Use Misuse. Author manuscript; Available in PMC 2020 Oct 21. (PMC7116233; doi:10.1080/10826084.2020.1725055)
Supplement: Supplementary materials [file EMS97872-supplement-Supplementary_materials.docx]

**Comprehensive Methods**

***Participants***

Participants for the study consisted of 114 male College students between the ages of 19 and 35 years (*M* = 24.89; *SD* = 2.54) recruited from the university town Nsukka in south-east Nigeria. Participants were included in one of three groups. Chronic marijuana users (*n* = 35; *M*_age_ = 25.40; *SD* = 2.53) self-reported to smoke at least five grams of marijuana per week (*M* = 10.40; *SD* = 6.04) and they reported not to drink more than four bottles of beer (or equivalent amounts of other alcoholic beverages) per week. Chronic alcohol drinkers (*n* = 39; *M*_age_ = 25.05; *SD* = 2.29) self-reported to drink at least five bottles of beer per week (*M* = 6.36; *SD* = 1.98) and not to smoke at least five grams of marijuana per week. Controls (*n* = 40; *M*_age_ = 24.20; *SD* = 2.68) self-reported to have abstained from alcohol use at least for the past one year, to have never smoked marijuana in their lifetime, and to have consumed no more than five bottles of alcoholic beverages in their lifetime. The three groups did not differ significantly with regard to age, *F*(2, 111) = 2.31, *p* = .104, and education, *F*(2, 111) = 1.83, *p* = .166. All participants were asked to strictly abstain from alcohol and marijuana at least 12 hours before the study participation. This is to ensure that the participants were not intoxicated during the neuropsychological examination. We were not able to carry out urine analyses to ensure compliance with this directive. Participants were excluded when they met one of the following criteria: 1. use of any other illicit or prescription drug common among youth in the sampled country including codeine, tramadol, rohypnol (refnol) within the past year; 2. current or past use of any psychotropic medication; 3. history of substance use disorders for first degree family relatives; 4. history of any medical or neurological disorder that interferes with cognitive functions including epilepsy and Attention Deficit Hyperactive Disorders; 5. previous head injury leading to loss of consciousness or seizures; 6. lack of understanding of basic English. All procedures were in accordance with the Declaration of Helsinki and approved by the local ethics committee.

***Procedures and assessment***

Students were approached and tested using a 14inch laptop at different places in the university town of Nsukka. Recruitment and contact sites included University campuses, colleges, student residential areas, concentrated drug zones and alcohol bars, sports and recreational centres. In addition, participation invitations were sent out via students’ social media platforms. When recruitment sites were not conducive for data collection, participants were booked for an appointment in the neurophysiology laboratory of the Department of Psychology, University of Nigeria, Nsukka. Irrespective of the testing site, we ensured a testing room that was similar to the lab with regard to comfort, ventilation, lighting conditions, silence and absence of distractions. Control participants were easily recruited through invitations on students’ social media and visits to classrooms and hostels. To recruit participants for the groups of alcohol and marijuana users, we primarily relied on snow-ball sampling (i.e., asking already recruited participants to refer other drug users to our study). This is because drug use is highly restricted in Nigeria and we realized that many drug users were only willing to participate when being referred to us by someone they know. At the testing room which was either a library or laboratory within the University, the aims and objectives of the study were explained in detail in a consent form, which each participant signed prior to participation. Participants were informed that participation was voluntary, that they can suspend their participation in the course of the assessment and that they would not be reimbursed for their participation.

All participants completed a computerized version of the WCST (cWCST; Lange, Kröger, et al., 2016; Lange, Seer, Dengler, Dressler, & Kopp, 2016; Lange, Seer, Finke, Dengler, & Kopp, 2015). The task version used in the present study has been designed in OpenSesame version 3.1.4 (Mathôt, Schreij, & Theeuwes, 2012), is described in detail elsewhere (Lange & Dewitte, 2019), and can be downloaded at https://osf.io/5t6fs/. On each trial of the cWCST, participants were required to match a target card to one of four key cards according to one of three possible sorting rules (colour, shape, number). Cards were sorted by pressing one of four keys that were mapped to the spatial position of the key cards on the screen. Card sorts were followed by a feedback cue that indicated whether the applied sorting rule should be maintained (“REPEAT”) or changed (“SWITCH”) on the next trial. Rules changed after runs of two or more rule repetitions. Participants completed six practice runs and 42 task runs or a maximum of 250 trials, whichever occurred first.

Accuracy and latency of cWCST sorting responses were quantified separately for three different trial types. The first trial after a change in task rules is referred as *switch trial*. On this trial, participants have to disengage from a previously correct rule and to switch to one of the two remaining rule. Failures to do so result in *perseverative errors*. When participants switch rules on the switch trial but do not switch to the correct rule, they encounter an *integration trial*. Here, they need to integrate information from the previous two trials in order to infer the correct new rule. Failures to do so result in *integration errors*. The first trial after the correct new rule has been identified (as signalled by a “REPEAT” feedback cue) is referred to as *repetition trial*. On this trial, participants need to apply the same rule that they successfully applied on the previous trial. Failures to do so result in *set-loss errors*. Perseverative, integration, and set-loss errors are considered to indicate deficits in set shifting, rule inference, and set maintenance (Lange et al., 2017).

***Data analysis***

We calculated the proportion of perseverative errors, integration errors, and set-loss errors as well as mean response times for successful rule switches on switch trials, successful integrations on integration trials, and successful rule repetitions on repetition trials. Response times below 200 ms or more than three standard deviation above an individual’s average were excluded for the calculation of mean response time. cWCST performance measures were compared between groups using mixed ANOVAs implemented in SPSS. By this means, it was possible to not only examine whether groups of alcohol users, marijuana users, and controls differed in overall cWCST performance (as indicated by overall group effects), but also whether group differences are specific to a particular cWCST performance measure (as indicated by an interaction between group and trial/error type). The level of significance was set at α = .05.

**References**

Lange, F., & Dewitte, S. (in press). Cognitive Flexibility and Pro-environmental Behavior: A Multimethod Approach. *European Journal of Personality*.

Lange, F., Kröger, B., Steinke, A., Seer, C., Dengler, R., & Kopp, B. (2016). Decomposing card-sorting performance: Effects of working memory load and age-related changes. Neuropsychology, 30(5), 579-590. doi:10.1037/neu0000271

Lange, F., Seer, C., Finke, M., Dengler, R., & Kopp, B. (2015). Dual routes to cortical orienting responses: Novelty detection and uncertainty reduction. Biological Psychology, 105, 66-71. doi:10.1016/j.biopsycho.2015.01.001

Lange, F., Seer, C., Dengler, R., Dressler, D., & Kopp, B. (2016). Cognitive Flexibility in Primary Dystonia. Journal of the International Neuropsychological Society, 22(6), 662-670. doi:10.1017/s135561771600045x

Lange, F., Seer, C., & Kopp, B. (2017). Cognitive flexibility in neurological disorders: Cognitive components and event-related potentials. Neuroscience & Biobehavioral Reviews, 83, 496-507. doi:10.1016/j.neubiorev.2017.09.011

Mathôt, S., Schreij, D., & Theeuwes, J. (2011). OpenSesame: An open-source, graphical experiment builder for the social sciences. Behavior Research Methods, 44(2), 314-324. doi:10.3758/s13428-011-0168-7
